# 

**Published:** 1897-02

**Authors:** 


### CONTENTS:

### SELECTIONS.

Article I.—Dentistry in the Orient. By Richard Henry Kimball, D. D. S. - - - - - - - - 433

" II.—Taking Impressions and Fitting Artificial Dentures. By Dr. W. T. Magill. - - - - - - 446

" III.—Pothology and Therapeutics of Dead Teeth. By Dr. G. H. Claude. - - - - - - - - 449

" IV.—The Investigation of Uric Acid in Salivary Tartar in the Course of Pyorrhea Alveolaris (Artaro-Dental Infectious Gingivitis). By Dr. Galippe. - 452

" V.—Eucain. By Dr. J. C. Clemesha, M. D., M. R. C. S., Eng., L. R C. P.. Lond. - - - - - 455

" VI.—Oral Surgery. By Edmund W. Roughton, B. S., M. D., Lond., F. R. C S., Eng. - - - - - 458

" VII.—A History of Maryland's New Dental Law, 1896. By Richard Grady, M. D., D. D. S. - - - 463

" VIII.—The Bicycle and the Perineum. - - - - - 471

### EDITORIAL, ETC.

Hygiene of the Mouth. - - - - - - - - 473

### MONTHLY SUMMARY.

A Case of Pyorrhea Alveolaris. - - - - - - - - 476

Metal Dies Direct from Impression. - - - - - - 476

Dental Sinners. - - - - - - - - - - 477

A New Test for Albumen. - - - - - - - - 478

Longevity of Human Life Increasing. - - - - - - 478

Liquid Silex. - - - - - - - - - - 479

Attachment of Logan Crown. - - - - - - - 479

Itching in Eczema. - - - - - - - - - 479

A New Hemostatic. - - - - - - - - - 480

Ice to Relieve Dyspepsia. - - - - - - - - 480

Another Source of Infection. - - - - - - - 480

